# Induced Dipoles and Possible Modulation of Wireless Effects in Implanted Electrodes. Effects of Implanting Insulated Electrodes on an Animal Test to Screen Antidepressant Activity

**DOI:** 10.3390/jcm10174003

**Published:** 2021-09-04

**Authors:** Laura Perez-Caballero, Hector Carceller, Juan Nacher, Vicent Teruel-Marti, Eulalia Pujades, Nieves Casañ-Pastor, Esther Berrocoso

**Affiliations:** 1 Neuropsychopharmacology and Psychobiology Research Group, Department of Psychology, University of Cádiz, 11003 Cádiz, Spain; laura.perez@uca.es; 2 Instituto de Investigación e Innovación Biomédica de Cádiz, Hospital Universitario Puerta del Mar, 11009 Cádiz, Spain; 3 Centro de Investigación Biomédica en Red de Salud Mental (CIBERSAM), Instituto de Salud Carlos III, 28029 Madrid, Spain; hector.carceller@uv.es (H.C.); Juan.Nacher@uv.es (J.N.); 4 Neurobiology Unit, Institute for Biotechnology and Biomedicine (BIOTECMED), Universitat de València, 46100 Valencia, Spain; 5 Fundación Investigación Hospital Clínico de Valencia, INCLIVA, 46010 Valencia, Spain; 6 Neuronal Circuits Laboratory, Department of Human Anatomy and Embryology, University of Valencia, 46010 Valencia, Spain; vicent.teruel@uv.es; 7 Institut de Ciencia de Materials de Barcelona, CSIC, Campus UAB, 08193 Barcelona, Spain; epujades@icmab.es

**Keywords:** Deep Brain Stimulation, depression, infralimbic cortex, rat, induced dipoles, implanted materials, feedback interactions, insulating, conducting materials

## Abstract

There is evidence that Deep Brain Stimulation (DBS) produces health benefits in patients even before initiating stimulation. Furthermore, DBS electrode insertion in rat infralimbic cortex (ILC) provokes antidepressant-like effects before stimulation, due to local inflammation and astrogliosis. Consequently, a significant effect of implanting electrodes is suspected. External fields, similar in magnitude to the brain’s endogenous fields, induce electric dipoles in conducting materials, in turn influencing neural cell growth through wireless effects. To elucidate if such dipoles influence depressive-like behavior, without external stimulation, the comparative effect of conducting and insulated electrodes along with the glial response is studied in unstressed rats. Naïve and implanted rats with electrically insulated or uninsulated steel electrodes were evaluated in the modified forced swimming test and expression of ILC-glial markers was assessed. An antidepressant-like effect was observed with conducting but not with insulated electrodes. Gliosis was detected in both groups, but astroglial reactivity was larger near uninsulated electrodes. Thus, induced dipoles and antidepressant-like effects were only observed with conducting implants. Such correlation suggests that dipoles induced in electrodes by endogenous fields in turn induce neuron stimulation in a feedback loop between electrodes and neural system. Further research of the effects of unwired conducting implants could open new approaches to regulating neuronal function, and possibly treat neurological disorders.

## 1. Introduction

Electrostimulation using implanted electrodes with alternating electric fields of low intensity is known to eliminate symptoms in Parkinson’s disease, dystonia and essential tremor or epilepsy. In Deep Brain Stimulation (DBS), stimulation electrodes are permanently connected to a neurostimulator with the capability of delivering electrical chronic stimulation of a targeted brain region. The extensive experience using this technique reveals that is a safe and well-tolerated therapy [1,2,3]. The effectiveness demonstrated in these neurological disorders led to the exploration of this approach for managing psychiatric diseases, such as major depressive disorder [4,5,6].

The fundamental mechanisms by which external alternating electric fields have such effect are not well known. However, there is evidence that the presence of continuous fields has intense effects on neurons in vitro including increased differentiation, directional growth of neurites towards the cathode, increased neural branching and filopodial activity [7]. Moreover, several studies have demonstrated that DBS can induce neuroplastic changes in the adult brain, such as alterations in the expression of molecules related to synaptic transmission [8,9,10,11,12,13]. On the other hand, little focus is given in general to the electrode materials in electrostimulation work. However, the interaction between the required electrodes and neurons is starting to show that the effects are highly dependent on the electrode material.

Recent significant work in electroactive biocompatible materials and neuroscience offers suggestive findings on the biophysical details that work when electric fields are present in the nervous system. Very significant is the observation that the simple implantation of the electrodes already induces a short-term effect, as mentioned above. Strikingly, an initial beneficial effect when using DBS has been reported in patients suffering from several neurobiological diseases, such as Parkinson’s disease, epilepsy or depression [14,15,16,17,18,19,20]. In animal models, electrode insertion into the infralimbic cortex (ILC; the rodent equivalent to human Cg25) is enough to provoke an antidepressant-like effect over a limited period (established after 1 week and lost by 6 weeks), without external electrical impulse [21]. Furthermore, this effect is accompanied by a local decrease in glucose metabolism and changes in brain areas commonly related to depression and the antidepressant response [22]. Significantly, when the electrodes were inserted in other areas of the medial prefrontal cortex, such as the prelimbic cortex or cingulate cortex, the antidepressant-like effect was not found [21]. Thus, it appears that the short-term amelioration of depressive symptoms following DBS surgery might reflect a physiological response to a set of steel electrodes insulated in the main body axis with only the tip being exposed to the biological electrolyte in very defined brain positions.

Interestingly, a fruitful simultaneous/coincidental work came about the same year, in which unwired electrode materials being used as a substrate for directional growth of *Xenopus* neural cells [23] have offered a breakthrough in the possible interaction between conducting materials and nervous system cells and suggest a different view. In the presence of electric fields (either imposed or endogenous fields), the classical physics theory in infinite systems says that the inner electric field is zero in the material. However, the real systems have spatial limits, and the boundaries of the conducting material, right where metallic conductivity disappears, undergo the formation of a dipole, opposing the direction of the imposed field. In the presence of large enough induced potentials, these dipoles generate electrochemical reactions at the respective anode and cathode in the unconnected material, in what is usually called bipolar electrochemistry [23]. Those reactions may be related to the aqueous electrolyte (H_2_ and O_2_ production at induced cathode and anode of the material, respectively), or to the material itself. Thus, copper gets oxidized forming CuO at the induced anode, steel may form iron oxides, and intercalation materials such as iridium oxide may undergo redox intercalation processes (Na^+^ intercalating in the induced cathode of the IrO_x_ material, for example). In addition, such a dipolar effect modifies the redox potential of the material in an inhomogeneous way, creating a redox spatial gradient, and therefore a directional chemical gradient. It can also vary the concentration of ions near one of the poles of the implanted material, while the other pole has a different ionic or redox species [24]. Thus, neural cells grown on conductive materials show varying speeds and directions of growth if an external field is applied [23]. Furthermore, it has been observed recently that the derived electrochemistry at the induced poles has effects during days and weeks [24], on a time scale similar to the antidepressant-like effect described in rats [21]. Such long-term effects on the materials are related to the gradient created due to oxidation, or intercalation of ions at the induced anode or cathode, respectively, and are described in detail in [24].

When attempts are made to evaluate the magnitude of such dipoles, wires are connected to a multimeter and the dipole discharges, and no voltage is measured. However, the dipole can be observed through optical changes, Energy Dispersive X-ray Spectroscopy (EDX) analysis, or X-ray absorption spectroscopy (XAS) measurements with spatial resolution and estimated by specific voltage measurements [25]. In that sense, the observation that turning the electric field off induces a disappearance of previous electrostimulation effects must be taken carefully. If connected wires still exist, the dipole in the implant is not seen.

Joining both observations [21,23], with the unexpected antidepressant-like effect with simple electrode implantation and the bipolar electrochemistry effects on materials directing neuron growth, the possibility of electrical interactions between neurons and the implanted material gained a new perspective. Thus, it is possible that the significant dynamic fields present endogenously in an alive nervous system (40 to 400 mV/mm as reported in [7]) induce dipoles (possibly dynamic also) in any implanted conducting material. Accordingly, it is also plausible that the material may in turn offer a feedback stimulation on nearby neurons, due to the dipole generated among its poles. Indeed, the electric fields where bipolar effects have been observed are in the same order of magnitude or lower [23]; 50 mV/mm shows effects in the intercalation materials, while 150 mV/mm induces H_2_ and O_2_ formation as well as pH changes [23].

With this idea in mind, it is a fundamental question to define whether the spontaneous activation observed is due to the possible generation of induced dipoles and therefore to the conducting character of the material. This work tackles just the first step to answer the question, by comparing in unstressed animals the response of in vivo activity and inflammation aspects for conducting implants and insulated implanted materials where no induced dipole can appear, restricting the case to the same dimensions and shape, and in the same zone of the brain.

For this purpose, a very thin coating with an insulating layer of similar hydrophilicity and expected proinflammatory effects is used on the standard electrodes. Similar hydrophilicity involves similar acid–base and chemical interactions in the neutral pH found in the nervous system. The criteria for comparison was the same as the one followed in previous observations where behavior patterns based on mobility and activity were tackled. Animals with insulated or non-insulated electrodes implanted were submitted to the modified forced swimming test to evaluate depressive-like behavior. An additional study includes the possible difference in inflammation effects in the nearby tissue, for both insulating and conducting materials, as it is the first suspected effect.

If differentiation upon implantation of conducting and insulating materials is possible, induced dipoles due to electrical interactions gain a new place in the biophysical explanations of material–cell interactions and open a new path to establish additional factors to be tackled in the future, for possible long term clinical applications through wireless stimulation, but also to deepen in the fundamental knowledge of material–cell interactions.

## 2. Materials and Methods

### 2.1. Materials Implanted and Characterization

The bipolar uninsulated electrodes used were two stainless steel enamel-coated wires (150 µm diameter: 304 type, California Fine Wire Co., Grover Beach, CA, USA) with a 1 mm exposed tip, while the insulated electrodes were the same but with the tip coated with dental acrylic cement (TAB2000, Kerr-Dental, Madrid, Spain).

Hydrophilicity measurements were performed through contact angle measurements using a Drop Shape Analyzer DSA 100 (Kruss Scientific, Hamburg, Germany) with drops of 8 μL using steel, but in larger pieces and flat-required configurations an equivalent alloy (Stainless Steel ALSi 316, Goodfellow, Coraopolis, PA, USA) and the TAB 2000 Kerr dental cement made as a 1 mm approximate coating was used, since such measurements require a flat surface not possible with the geometry of implanted electrodes. Both distilled Milli-Q water and 0.1 M sodium phosphate buffer, pH 7.4 were used as a plain solvent and ionic solutions.

### 2.2. Induced Dipoles and Bipolar Electrochemistry Visualization

Dipoles induced in conducting materials as compared to no dipole cases in insulated electrodes were visualized here for 10 V/2.4 cm external fields, immersing both pieces between Pt parallel driving electrodes. A redox color indicator was used for better visualization, a 0.1 M [SiW_12_O_40_]^−4^ aqueous solution that reduces to [SiW_12_O_40_]^−6^ (deep blue) at the induced cathode in steel, but not on insulated steel. Dipoles also exist in weaker fields [50 mV/mm] as reported before [23].

### 2.3. Animals

Male Wistar rats (University of Cadiz, weighing 250–300 g at the beginning of the experiments) were housed under standard laboratory conditions (22 °C, 12 h light/dark cycle, food and water ad libitum). All the procedures followed were approved by the Animal Research Ethics Committee (University of Cádiz, PI12-00915, 2012), and they were in accordance with European-Guidelines (2010/63/EU) and Spanish Law (RD 53/2013).

### 2.4. Experimental Design

**Figure 1:**
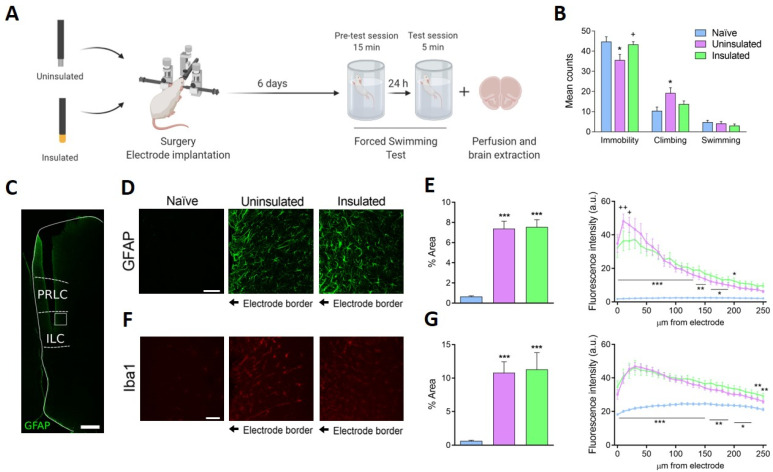
Insulation of DBS electrodes blocks the antidepressant-like effect evident in the modified forced swimming test and it affects glial reactivity in the vicinity of the implant. (**A**) Scheme illustrating the experimental timeline. (**B**) The graph represents the mean counts of immobility, climbing and swimming behavior at 5-s intervals in the modified forced swimming test (mFST: immobility F_(2,25)_ = 4.69, *p* < 0.05; climbing F_(2,25)_ = 4.40, *p* < 0.05; swimming F_(2,25)_ = 0.93, *p* > 0.05). (**C**) Low magnification confocal single image showing a representative trace of an implanted electrode. The white stroke square in the ILC shows the area imaged and quantified. Scale bar, 500 µm. (**D**) Representative confocal images of glial fibrillary acidic protein (GFAP) staining in the different experimental groups. (**E**) The plots show the proportion of the confocal image in which the GFAP astrocyte marker is detected (left panel: W_(2,10.18)_ = 80.27, *p* < 0.001) and the variation in the fluorescence intensity at the edge of the implant (right panel: interaction F_(50, 520)_ = 6.37, *p* < 0.001). (**F**) Representative confocal images of immunostaining for the ionized calcium-binding adapter molecule (Iba1) in the different experimental groups. (**G**) The plots show the proportion of the confocal image in which the microglial marker Iba1 was detected (left panel: F_(2,9.99)_ = 27.02, *p* < 0.001) and the variation in fluorescence intensity from the edge of the implant (right panel: interaction F_(50, 520)_ = 3.27, *p* < 0.001). Scale bar, 40 µm. The values are the means ± SEM, analyzed with a one-way (ordinary in 2B and Welch’s corrected in left panels of 2E and 2G) or two-way ANOVA followed by a Newman–Keuls post-hoc test: * *p* < 0.05, ** *p* < 0.01, *** *p* < 0.001 versus naïve rats; + *p* < 0.05, ++, *p* < 0.01 versus uninsulated DBS-off rats. The asterisks below a line indicate that both groups showed significant differences (*n* = 7–9 per group). ILC: infralimbic cortex, PRLC: prelimbic cortex.

The electrodes were implanted into the ILC of unstressed animals and 1 week later animals were assessed in the modified forced swimming test (mFST), and they were sacrificed immediately after for immunofluorescence studies. For histology, one brain of naïve group and one brain of implanted uninsulated electrodes were removed due to poor fixation. Three groups of rats were studied: naïve rats (*n* = 8 in behavior and 7 in histology) and rats implanted with uninsulated (*n* = 9 in behavior and 8 in histology) or insulated electrodes (*n* = 9, Figure 1A).

### 2.5. Surgery

Rats were anesthetized with a ketamine/xylazine mixture (100 mg/kg/12 mg/kg, i.p.) and positioned in the stereotaxic apparatus (Kopf Instruments, Tujunga, CA, USA). The electrodes were implanted bilaterally into the ILC (AP + 3.2 mm from bregma; L ± 0.5 mm; DV −4.3 mm from dura mater) [26] via a sagittal incision made to expose the skull and with holes drilled with a bur connected to a micromotor (Namrol). The electrodes were then fixed to three stainless steel screws secured to the skull using dental acrylic cement (Proclinic SA, Barcelona, Spain).

### 2.6. mFST

The mFST is one of the most widely used preclinical tools to study antidepressant activity in rats [27]. One week after implantation, rats were placed in clear Plexiglas cylinders (height 50 cm; diameter 20 cm) filled to a depth of 30 cm with water at 25 ± 1 °C, initially for 15 min in the first swimming (pre-test) session and then, for 5 min 24 h later (test session). The tests were recorded and subsequently scored by a highly trained observer, who was blind to the treatment. Behavior was assessed at 5-s intervals throughout the duration of the test session using customized software (Red Mice, Cádiz, Spain). After each 5 s interval, the predominant behavior was assigned to one of three categories, immobility, swimming or climbing. Climbing was defined as forceful thrashing movements of the forelimbs directed against the walls of the cylinder. Swimming was defined as moving all four paws in an active swimming motion that was more vigorous than is necessary to merely maintain their head above water. Immobility was noted when the rat remained floating in the water without struggling, only making movements that were required to keep it afloat. Reduced immobility was considered to indicate anti-depressant activity [27].

### 2.7. Immunohistochemistry

Animals were perfused with 4% paraformaldehyde in phosphate buffer (PB, pH 7.4). Brains were cryoprotected for 24 h by immersion in 30% sucrose dilution in PB. Then, coronal sections (50 μm) were obtained using a freezing microtome (Leica, Wetzlar, Germany). Sections were processed for immunohistochemistry using chicken anti-GFAP (Abcam, Cambridge, UK, ab4674, 1:1000) and rabbit anti-Iba1 (Abcam, ab178846, 1:2000) primary antibodies, as described previously [28]. The position of electrodes was checked under a fluorescence microscope (Olympus BX41, Olympus, Tokyo, Japan). All the animals were correctly implanted in the ILC; thus they were included in the quantification of glial markers. We imaged one coronal section per animal where the electrodes trace was clearly visible in the ILC region (between 2.70 and 3.00 mm from Bregma), according to rat brain atlas [26], and we used a 20× objective plus 2× digital zoom in the confocal microscope (Leica SPE, Leica Microsystems, Wetzlar, Germany) to acquire confocal stacks of the whole section. The acquiring parameters were maintained throughout all the imaging sessions to compare the fluorescence intensity between samples.

Fluorescence intensity relative to the distance from the electrode was then quantified in confocal microscopy images of the tissue around the trace and analyzed with Fiji [29] using the plot profile tool. For the quantification of the area labeled by glial markers, we used a set of previously validated custom-made macros [30].

### 2.8. Statistical Analysis

All the analysis and graphs were obtained using Graphpad Prism 9.0 software (GraphPad Software, San Diego, CA, USA). The data are presented as the mean ± S.E.M. Normality and homoscedasticity of data were evaluated in order to conduct the appropriate statistical test. One- (ordinary or Welch’s corrected) or two-way ANOVA followed by Newman-Keuls post-hoc analyses were used to assess significance (accepted at *p* < 0.05).

## 3. Results

### 3.1. Visualization of Bipolar Electrochemistry Effects Due to Induced Dipoles in Unwired Implants

**Figure 2:**
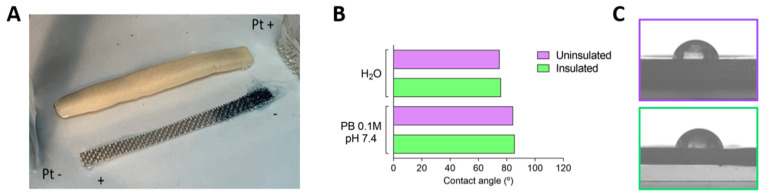
Dipole and bipolar electrochemistry effect and hydrophilicity. (**A**) Bipolar electrochemical effects on the steel electrode immersed in a solution with a redox indicator [SiW_12_O_40_]^−4^, (0.1M in water) relative to the same material coated with dental cement. External driving electrodes Pt 99.9%, applied external voltage 9 V. (**B**) Contact angle values of uninsulated and insulated electrode tips in pure distilled water and phosphate buffer (PB 0.1M, pH 7.4). (**C**) Representative images of hydrophilicity of uninsulated and insulted electrode tips in PB 0.1M, pH 7.4.

The existence of external (or endogenous) electric fields [7] generates a dipole on conducting implanted materials that may yield electrochemical reactions at the induced anode and cathode in such material [23,31]. Figure 2A shows an example of such a bipolar process where a cluster anion, [SiW_12_O_40_]^−4^, gets reduced at the induced cathode in stainless steel, yielding [SiW_12_O_40_]^−6^, dark blue in color. Such reduction process does not occur for insulated steel, evidencing that no dipole is induced. Larger voltages yield an additional reaction in the poles of the conducting material, the formation of H_2_ in the induced cathode, and O_2_ in the opposite induced anode. It is worth noting here that the external applied fields using Pt electrodes are similar to those that endogenous fields from alive neurons can establish in animals [7].

Figure 2B,C also shows that the hydrophilicity of both surfaces, steel and dental cement, have similar values, both in water or in a phosphate buffer. This similar interaction with aqueous systems also suggests a similar interaction with the brain tissue.

### 3.2. Antidepressant-Like Effect

A significant reduction in the time spent immobile in mFST was evident in the uninsulated rats compared to the naïve and insulated animals. This reduction in immobility was accompanied by an increase in climbing behavior, while no significant differences in swimming behavior were observed. Strikingly, animals that were implanted with insulated electrodes did not exhibit significant differences in any of the behaviors, compared to naïve animals (Figure 1B).

### 3.3. Glial Activation in the ILC

As expected, implantation of the electrodes augmented gliosis (Figure 1C) produced a larger area stained for the astrocyte marker GFAP in the rats that received uninsulated and insulated electrodes, with no differences between these two groups. However, when astrocyte activation was evaluated in relation to the distance from the electrode (fluorescence intensity within the 250 μm surrounding the implant), uninsulated electrodes produced more intense GFAP immunofluorescence than insulated electrodes 10 and 20 μm from the border of the implant (Figure 1D,E). Electrode implantation also increased microglia labeling, witnessed by a similar increase in Iba1 immunoreactivity in uninsulated and insulated groups, although electrode insulation did not produce any difference in microglia. Furthermore, similar results were found in both groups when the distance from the electrode was taken into consideration (Figure 1F,G).

## 4. Discussion

The observations described above evidence that the simple implantation of a conducting material in the ILC can produce an antidepressant-like effect in unstressed rats, as reported before [21], and that such effect is blocked when the conducting material is insulated.

As previously reported for other materials [23,24,25], steel electrodes also get polarized when immersed in an ionic media, in the presence of external applied fields, yielding to electrochemical effects that involve oxidation in the induced anode pole and reduction in the opposite cathode pole, at certain external applied potentials. That polarization does not occur when the electrode is protected with an insulating layer. The magnitude of endogenous electric fields in biosystems [7] is similar to those used in reported experiments [23,24] and therefore could induce the same polarization in the implanted material. Of course, endogenous fields possess a dynamic character which is not included in the previous experiments [23]. Yet, at some short scale time, the external field effects may be assimilated, as is similar to the possible field effects that endogenous fields from neurons may have on conducting implanted materials, but not for insulating materials. Therefore, it is plausible that the nervous system generates induced dipoles in implanted conducting materials, and not on insulating materials.

Indeed, a significant correlation is found in this work between biological observations and the conducting character or its absence for the implanted material. Implants with conducting (uncoated) steel electrodes induce antidepressant-like effects, evident 1 week after electrode implantation and lost by 6 weeks. Moreover, this effect was of similar magnitude to that elicited by DBS stimulation [21]. Strikingly, this antidepressant-like reaction is lost when the tip of the electrode is insulated with dental clay, suggesting a beneficial interaction between the conducting material and the neural tissue exposed.

In the ILC, uninsulated electrodes also induce several changes related to antidepressant-like behavior, such as local decreases in glucose metabolism or the glial response in the nearby tissue [21,22]. Reactive gliosis is involved in the remodeling and reshaping of neural circuits during healing and the therapeutic effects of neuromodulation [32,33]. Thus, although a similar increase in microglia labeling was evident in both groups, a significant increase in reactive was found in the area surrounding the uninsulated conducting electrode relative to the insulated one. This is consistent with our previous data showing a temporal correlation between the behavioral antidepressant-like effects and the time required for glial scar formation [21]. Furthermore, non-steroid anti-inflammatory drugs (NSAIDs) reduce GFAP expression and block the antidepressant-like effect of the uninsulated electrode [21,22], reinforcing the conclusion that astrocytes are effectors of stimulation-based therapy [32]. Nevertheless, future studies comparing the effects of insulated and uninsulated electrodes using NSAIDs will be necessary to completely understand the role of gliosis on the effects of induced dipoles.

If electric perturbations of the endogenous electric fields exist, a possible physical cause of behavior change could be related to such a fact. However, indeed, we may consider that the observed effects on behavior for animals with the implanted unwired electrodes may also be due to different inflammatory effects and neural activation mechanisms related to the surface chemistry. In addition to the expected inflammation factors related to surgery and implantation that may induce glial activations and modify interneuronal connectivity, we must include as possible responsible factors the electrochemical processes that may occur at the dipoles generated by endogenous fields (water oxidation at the anode forming O_2_ or Oxygen radicals, and dissolved O_2_ reduction to form oxygen radicals at the induced cathode).

Thus, electric interactions in DBS may therefore be simply physical, and endogenous fields may induce dipoles in the conducting electrode, in a feedback loop that could explain possible interactions. However, the existence of polarization demonstrated before also shows chemical and electrochemical processes that may affect gliosis and inflammation.

Ideally, other materials with different surface chemistries could be tested in vivo, as they have been in vitro. More than a dozen materials have been tested before in Xenopus neural cells culture models with low density of neurons, in absence of external electric stimulation (either wired or unwired) [23]. In most biocompatible cases, in absence of fields, many different chemistries have shown a good neural adhesion and neurite development, and only a few examples prevent neural growth, possibly from hidden incompatible surface chemistry. No significant inflammatory differences appear among all those materials when mammalian cells are used, for cases where that has been tested [34,35]. In the presence of connected electrostimulation electrodes, scratch repair models show that the neurons react differently depending on the material [35]. However, in the presence of external fields, for unwired electrodes, the induced dipoles exist for all conducting materials, and modify substantially the speed and direction of Xenopus neurite growth, differentiating the behavior of cells clearly from that found on insulating materials.

While a few cultured cells are not able to create global endogenous fields similar to the in vivo fields, the external imposed fields may simulate such values in culture models, and therefore Xenopus wireless effects represent a good model to find parallelism with this work.

What is shown above is a first step toward elucidating if the induced dipoles may be the responsible ones (both physical and chemical aspects), of the behavior and glial effects when a conducting material is implanted. Additionally, the comparison with an implant of the same geometry but insulated constitutes an empirical check of the influence of the electric character of the implant, with all variables involved.

In this line of thought, the observations described suggest that the induced dipoles and derived electrochemistry are the main known difference in antidepressant behavior with conducting or insulated materials implanted. Moreover, studies on gliosis and related neuroinflammation show differences, especially within the short-range distances from the electrode material, which could be related to those electrochemical processes in addition to the implantation and surgery effects.

## 5. Conclusions

Even though this is a preliminary study, our results suggest a significant difference between cases where induced dipoles exist and those where they are not possible. A clear and evident difference is observed in the depressive-like behavior of rats depending on the conducting character of the implanted material.

A difference in the neuroinflammatory response upon implantation was suspected to be responsible for the effect, but the observations show small changes which seem to not account for the different behavior. Differences observed in tissue near the implanted electrodes could be related to the dipole existence, known to exist in that same spatial range, and to the electrochemical possible reactions at each pole. Additional studies using depression models are indeed required, but a first step has shown a clear correlation between the difference of effects for conducting or insulated materials.

## References

[1] Fenoy A.J., Simpson R.K. (2014). Risks of common complications in deep brain stimulation surgery: Management and avoidance. J. Neurosurg..

[2] Kenney C., Simpson R., Hunter C., Ondo W., Almaguer M., Davidson A., Jankovic J. (2007). Short-term and long-term safety of deep brain stimulation in the treatment of movement disorders. J. Neurosurg..

[3] Krack P., Volkmann J., Tinkhauser G., Deuschl G. (2019). Deep Brain Stimulation in Movement Disorders: From Experimental Surgery to Evidence-Based Therapy. Mov. Disord..

[4] Clair A.H., Haynes W., Mallet L. (2018). Recent advances in deep brain stimulation in psychiatric disorders. F1000Res.

[5] Lee D.J., Lozano C.S., Dallapiazza R.F., Lozano A.M. (2019). Current and future directions of deep brain stimulation for neurological and psychiatric disorders. J. Neurosurg..

[6] Lozano A.M., Lipsman N., Bergman H., Brown P., Chabardes S., Chang J.W., Matthews K., McIntyre C.C., Schlaepfer T.E., Schulder M. (2019). Deep brain stimulation: Current challenges and future directions. Nat. Rev. Neurol..

[7] McCaig C.D., Rajnicek A.M. (1991). Electrical fields, nerve growth and nerve regeneration. Exp. Physiol..

[8] Casquero-Veiga M., Bueno-Fernandez C., Romero-Miguel D., Lamanna-Rama N., Nacher J., Desco M., Soto-Montenegro M.L. (2021). Exploratory study of the long-term footprint of deep brain stimulation on brain metabolism and neuroplasticity in an animal model of obesity. Sci. Rep..

[9] Chakravarty M.M., Hamani C., Martinez-Canabal A., Ellegood J., Laliberte C., Nobrega J.N., Sankar T., Lozano A.M., Frankland P.W., Lerch J.P. (2016). Deep brain stimulation of the ventromedial prefrontal cortex causes reorganization of neuronal processes and vasculature. Neuroimage.

[10] Jimenez-Sanchez L., Linge R., Campa L., Valdizan E.M., Pazos A., Diaz A., Adell A. (2016). Behavioral, neurochemical and molecular changes after acute deep brain stimulation of the infralimbic prefrontal cortex. Neuropharmacology.

[11] Shen K.Z., Zhu Z.T., Munhall A., Johnson S.W. (2003). Synaptic plasticity in rat subthalamic nucleus induced by high-frequency stimulation. Synapse.

[12] Tisch S., Rothwell J.C., Bhatia K.P., Quinn N., Zrinzo L., Jahanshahi M., Ashkan K., Hariz M., Limousin P. (2007). Pallidal stimulation modifies after-effects of paired associative stimulation on motor cortex excitability in primary generalised dystonia. Exp. Neurol..

[13] Veerakumar A., Challis C., Gupta P., Da J., Upadhyay A., Beck S.G., Berton O. (2014). Antidepressant-like effects of cortical deep brain stimulation coincide with pro-neuroplastic adaptations of serotonin systems. Biol. Psychiatry.

[14] Cersosimo M.G., Raina G.B., Benarroch E.E., Piedimonte F., Aleman G.G., Micheli F.E. (2009). Micro lesion effect of the globus pallidus internus and outcome with deep brain stimulation in patients with Parkinson disease and dystonia. Mov. Disord..

[15] Hodaie M., Wennberg R.A., Dostrovsky J.O., Lozano A.M. (2002). Chronic anterior thalamus stimulation for intractable epilepsy. Epilepsia.

[16] Lozano A.M., Mayberg H.S., Giacobbe P., Hamani C., Craddock R.C., Kennedy S.H. (2008). Subcallosal cingulate gyrus deep brain stimulation for treatment-resistant depression. Biol. Psychiatry.

[17] Mann J.M., Foote K.D., Garvan C.W., Fernandez H.H., Jacobson C.E., Rodriguez R.L., Haq I.U., Siddiqui M.S., Malaty I.A., Morishita T. (2009). Brain penetration effects of microelectrodes and DBS leads in STN or GPi. J. Neurol. Neurosurg. Psychiatry.

[18] Mayberg H.S., Lozano A.M., Voon V., McNeely H.E., Seminowicz D., Hamani C., Schwalb J.M., Kennedy S.H. (2005). Deep brain stimulation for treatment-resistant depression. Neuron.

[19] Morishita T., Foote K.D., Wu S.S., Jacobson C.E., Rodriguez R.L., Haq I.U., Siddiqui M.S., Malaty I.A., Hass C.J., Okun M.S. (2010). Brain penetration effects of microelectrodes and deep brain stimulation leads in ventral intermediate nucleus stimulation for essential tremor. J. Neurosurg..

[20] Tykocki T., Nauman P., Koziara H., Mandat T. (2013). Microlesion effect as a predictor of the effectiveness of subthalamic deep brain stimulation for Parkinson’s disease. Stereotact Funct. Neurosurg..

[21] Perez-Caballero L., Perez-Egea R., Romero-Grimaldi C., Puigdemont D., Molet J., Caso J.R., Mico J.A., Perez V., Leza J.C., Berrocoso E. (2014). Early responses to deep brain stimulation in depression are modulated by anti-inflammatory drugs. Mol. Psychiatry.

[22] Perez-Caballero L., Soto-Montenegro M.L., Hidalgo-Figueroa M., Mico J.A., Desco M., Berrocoso E. (2018). Deep brain stimulation electrode insertion and depression: Patterns of activity and modulation by analgesics. Brain Stimul..

[23] Rajnicek A.M., Zhao Z., Moral-Vico J., Cruz A.M., McCaig C.D., Casan-Pastor N. (2018). Controlling Nerve Growth with an Electric Field Induced Indirectly in Transparent Conductive Substrate Materials. Adv. Health Mater..

[24] Fuentes-Rodriguez L., Abad L., Simonelli L., Tonti D., Casañ-Pastor N. (2021). Iridium oxide redox gradient material: Operando X Ray absorption of Ir gradient oxidation states during IrOx bipolar electrochemistry. J. Phys. Chem. C.

[25] Fuentes-Rodriguez L., Abad L., Pujades E., Tonti D., Casañ-Pastor N. (2021). Induced dipoles and bipolar electrochemistry effects on electrolyte resistance. A macroscopic model experiment using immersed metal pieces. Electrochim. Acta.

[26] Paxinos G., Watson C. (2009). The Rat Brain in Stereotaxic Coordinates.

[27] Detke M.J., Rickels M., Lucki I. (1995). Active behaviors in the rat forced swimming test differentially produced by serotonergic and noradrenergic antidepressants. Psychopharmacology.

[28] Carceller H., Guirado R., Ripolles-Campos E., Teruel-Marti V., Nacher J. (2020). Perineuronal Nets Regulate the Inhibitory Perisomatic Input onto Parvalbumin Interneurons and gamma Activity in the Prefrontal Cortex. J. Neurosci..

[29] Schindelin J., Arganda-Carreras I., Frise E., Kaynig V., Longair M., Pietzsch T., Preibisch S., Rueden C., Saalfeld S., Schmid B. (2012). Fiji: An open-source platform for biological-image analysis. Nat. Methods.

[30] Guirado R., Carceller H., Castillo-Gomez E., Castren E., Nacher J. (2018). Automated analysis of images for molecular quantification in immunohistochemistry. Heliyon.

[31] Abad L., Rajnicek A., Casañ-Pastor N. (2019). Electric Field Gradients and Bipolar Electrochemistry effects on Neural Growth. A finite element study on inmersed electroactive conducting electrode materials. Electrochim. Acta.

[32] Salatino J.W., Ludwig K.A., Kozai T.D.Y., Purcell E.K. (2017). Glial responses to implanted electrodes in the brain. Nat. Biomed. Eng..

[33] Tawfik V.L., Chang S.Y., Hitti F.L., Roberts D.W., Leiter J.C., Jovanovic S., Lee K.H. (2010). Deep brain stimulation results in local glutamate and adenosine release: Investigation into the role of astrocytes. Neurosurgery.

[34] Lichtenstein M.P., Carretero N.M., Perez E., Pulido-Salgado M., Moral-Vico J., Sola C., Casan-Pastor N., Sunol C. (2018). Biosafety assessment of conducting nanostructured materials by using co-cultures of neurons and astrocytes. Neurotoxicology.

[35] Lichtenstein M.P., Pérez E., Ballesteros L., Suñol C., Casañ-Pastor N. (2017). Short term electrostimulation enhancing neural repair in vitro using large charge capacity intercalation electrodes. App. Mater. Today.

